# Real-World Outcomes of Lipoprotein Apheresis in Homozygous Familial Hypercholesterolemia: A Single-Center Longitudinal Experience

**DOI:** 10.3390/jcm15187233

**Published:** 2026-09-17

**Authors:** Filiz Mercan Sarıdaş, Canan Ersoy, Fahir Özkalemkaş, Erhan Hocaoğlu, Müge Yaşar, Kadircan Karatoprak, Özen Öz Gül, Soner Cander, Erdinç Ertürk

**Affiliations:** 1 Department of Internal Medicine, Division of Endocrinology and Metabolism, Faculty of Medicine, Bursa Uludağ University, 16059 Bursa, Türkiye; 2 Department of Internal Medicine, Division of Haematology, Faculty of Medicine, Bursa Uludağ University, 16059 Bursa, Türkiye

**Keywords:** homozygous familial hypercholesterolemia, lipoprotein apheresis, double-filtration plasmapheresis, therapeutic plasma exchange, LDL cholesterol

## Abstract

**Background:** Lipoprotein apheresis (LA) remains an important treatment option for patients with homozygous familial hypercholesterolemia (HoFH) who have persistently severe low- density lipoprotein cholesterol (LDL-C) elevation despite intensive lipid-lowering therapy. Therapeutic plasma exchange (TPE) may serve as a temporary alternative when selective LA is unavailable. **Methods:** This retrospective, single-center longitudinal observational study evaluated six patients with HoFH treated with double-filtration plasmapheresis (DFPP) between 2010 and 2023. During a temporary interruption in DFPP availability, two patients underwent TPE. Pre- and post-procedure lipid levels were evaluated using paired measurements. **Results:** A total of 476 DFPP and 20 TPE procedures were performed. During DFPP, the median reductions in total cholesterol and LDL-C were 64.44% [57.14–70.21] and 67.53% [59.53–73.86], respectively, while the median reductions in high-density lipoprotein cholesterol (HDL-C) and triglycerides were 40.00% [30.63–48.00] and 48.63% [35.38–57.66]. Among TPE procedures with paired measurements, the corresponding median reductions were 71.11% [65.52–72.69] for total cholesterol, 70.97% [67.14–73.72] for LDL-C, 67.08% [62.92–70.83] for HDL-C, and 55.73% [38.68–63.33] for triglycerides. No serious adverse events were observed during DFPP. TPE was discontinued in one patient after chills and subfebrile fever. **Conclusions:** Long-term DFPP was associated with substantial acute lipid reductions in patients with HoFH. TPE also produced marked acute lipid reductions in two patients when DFPP was unavailable. However, the limited number of TPE-treated patients, repeated procedures within the same individuals, and overlapping evolocumab exposure preclude conclusions regarding comparative efficacy or safety. TPE may be considered as an individualized interim option when selective lipoprotein apheresis is temporarily unavailable.

## 1. Introduction

Homozygous familial hypercholesterolemia (HoFH) is a rare inherited disorder characterized by markedly elevated low-density lipoprotein cholesterol (LDL-C) levels from birth and a substantially increased risk of premature atherosclerotic cardiovascular disease, aortic valve disease, and premature cardiovascular mortality. Contemporary estimates suggest a prevalence of approximately 1 in 300,000–400,000 individuals worldwide [1,2,3]. HoFH is most commonly caused by pathogenic variants affecting LDL receptor function, although variants involving apolipoprotein B (*APOB*), proprotein convertase subtilisin/kexin type 9 (*PCSK9*), and other genes may also contribute [3,4]. Without effective treatment, cardiovascular complications may develop early in life, making prompt diagnosis and intensive lifelong LDL-C lowering essential [1,3]. Current management generally requires combination therapy, including maximally tolerated statins and ezetimibe, PCSK9-directed therapies, lomitapide, evinacumab, and lipoprotein apheresis, with treatment individualized according to LDL-C burden, residual LDL receptor function, clinical status, and treatment availability [3,5,6,7].

Lipoprotein apheresis remains an important treatment option for patients with HoFH who have persistent severe LDL-C elevation despite intensive pharmacological therapy. Depending on the technique used, a single procedure can produce substantial acute reductions in LDL-C, while ongoing treatment at individualized intervals is required to maintain lipid control [5,8,9]. Double-filtration plasmapheresis (DFPP) is one of several selective apheresis techniques used to remove apoB-containing lipoproteins while limiting the removal of other plasma constituents [5,10,11]. Long-term observational data suggest that regular lipoprotein apheresis may contribute to cardiovascular risk reduction in patients with severe familial hypercholesterolemia [12,13,14].

Therapeutic plasma exchange (TPE) is a nonselective plasma-based procedure that has historically been used in severe familial hypercholesterolemia when selective lipoprotein apheresis is unavailable or cannot be performed [15,16,17]. TPE is used across a broad range of hematologic, neurologic, renal, and autoimmune disorders [17]. Unlike selective lipoprotein apheresis, TPE removes a broad range of plasma constituents together with LDL-C, including HDL-C and plasma proteins, which limits its routine use. Nevertheless, TPE may provide a temporary therapeutic option in selected clinical circumstances when access to selective lipoprotein apheresis is interrupted [5,10,17].

The present study aimed to retrospectively characterize long-term DFPP treatment in patients with HoFH and to describe the biochemical response, treatment course, and safety observations associated with temporary TPE use during a period when DFPP was unavailable.

## 2. Methods

### 2.1. Study Design and Patient Selection

This retrospective, single-center longitudinal observational study evaluated patients who underwent therapeutic apheresis at the Adult Endocrinology Department between 2010 and 2023. Patients were identified through the hospital information system using the keyword “therapeutic lipid apheresis.”

Patients were eligible if they underwent apheresis because of markedly elevated LDL-C levels and had a clinical diagnosis consistent with HoFH. The diagnosis of HoFH was based on the overall clinical and biochemical phenotype, family history, assessment according to the Dutch Lipid Clinic Network criteria, and available genetic findings [18]. Patients who underwent apheresis for hypertriglyceridemia-related acute pancreatitis, patients with secondary causes of elevated LDL-C, and those with insufficient clinical data were excluded.

**Figure 1:**
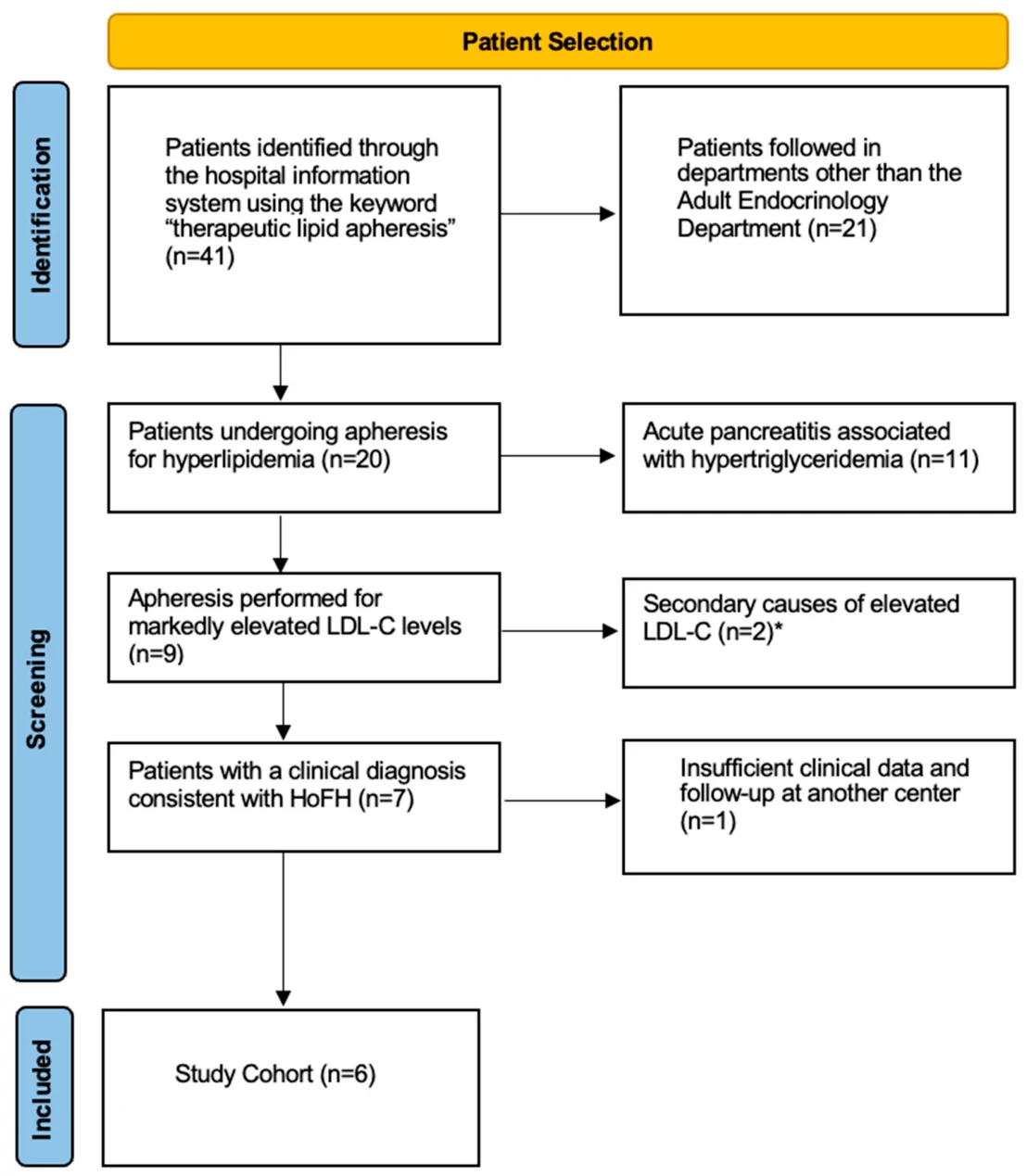
Flow chart of patient selection. * Hyperlipidemia secondary to liver cirrhosis.

Demographic and clinical data, including age, sex, age at diagnosis, age at treatment initiation, presenting symptoms, comorbidities, cardiovascular risk factors, lipid-lowering therapies, and available genetic test results, were retrospectively reviewed. All included patients had persistent severe hypercholesterolemia despite dietary measures and maximally tolerated lipid-lowering therapy. The patient selection process is summarized in Figure 1.

### 2.2. Apheresis Procedures

At our tertiary referral center, decisions regarding initiation and continuation of apheresis were individualized according to clinical characteristics, lipid levels, treatment response, and treatment availability, following discussion within the Endocrinology Council, which includes endocrinologists experienced in lipid disorders. DFPP was the standard lipoprotein apheresis modality used at our center. During the period when DFPP was unavailable, the temporary use of TPE in selected patients was additionally discussed with the Hematology team. Treatment frequency was individualized according to clinical requirements.

DFPP was performed using Medica (Medica S.p.A., Medolla, Italy) and Plasauto Σ (Asahi Kasei Medical Co., Tokyo, Japan) systems. Peripheral antecubital venous access was used, and heparin was administered as the anticoagulant. Procedures lasted approximately 2–2.5 h at a blood flow rate of 50–60 mL/min. No replacement solution was used during DFPP.

Between December 2021 and November 2022, DFPP was temporarily unavailable because of equipment and supply limitations. During this period, TPE with 5% human albumin was used as a temporary alternative in two patients. The sequential use of DFPP and TPE in these patients reflected temporary changes in treatment availability rather than a prespecified treatment-switching protocol. TPE was performed using the Spectra Optia system, version 7.2 (Terumo BCT, Lakewood, CO, USA), with peripheral venous access and acid citrate dextrose used for anticoagulation. Approximately 1.5 estimated plasma volumes were processed during each procedure. Procedures lasted approximately 2–2.5 h at a blood flow rate of 50–60 mL/min.

All patients received dietary treatment and maximally tolerated statin therapy. Ezetimibe was used in all patients except Patient 3, in whom it had been discontinued because of liver toxicity. Written informed consent for apheresis treatment was obtained before initiation of treatment.

### 2.3. Data Collection and Outcomes

Serum total cholesterol, LDL-C, HDL-C, and triglyceride levels obtained immediately before and after each apheresis procedure were recorded. For each lipid parameter, only sessions with paired pre- and post-procedure measurements were included in the corresponding biochemical analysis.

Percentage reduction for each lipid parameter was calculated as [(pre-procedure value − post-procedure value)/pre-procedure value] × 100. Missing biochemical values were not imputed. Concomitant lipid-lowering treatments, including evolocumab, were recorded according to treatment period to allow appropriate interpretation of the observed biochemical changes.

### 2.4. Ethics Approval

Our study was approved by the Bursa Uludağ University Faculty of Medicine Clinical Researchs Ethics Committee (approval no: 2023-27/29).

### 2.5. Statistical Analysis

Statistical analyses were performed using IBM SPSS Statistics, version 29.0.2.0 [20] (IBM Corp., Armonk, NY, USA). Given the small number of independent patients and the repeated nature of apheresis procedures within the same individuals, the statistical approach focused on descriptive summaries rather than inferential comparisons. Continuous variables were summarized as median and interquartile range [Q1–Q3], and categorical variables as number and percentage. For session-level biochemical analyses, only procedures with paired pre- and post-procedure measurements for the relevant lipid parameter were included. Percentage reduction was calculated as ((pre-procedure value − post-procedure value)/pre-procedure value) × 100. Because repeated procedures within individual patients were not statistically independent and TPE was administered to only two patients, no formal inferential comparison between DFPP and TPE was performed. Missing biochemical values were not imputed.

### 2.6. Use of Generative AI

**Figure 2:**
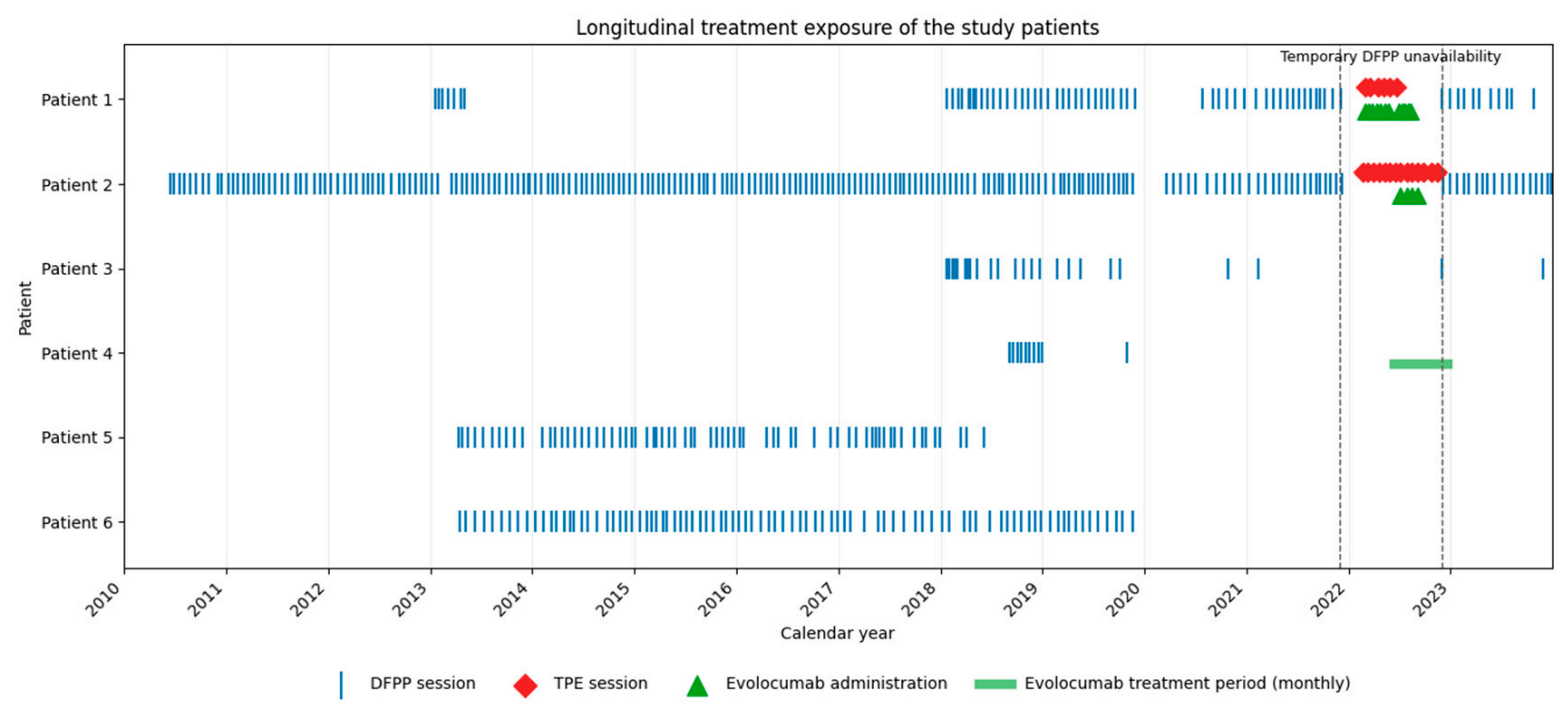
Longitudinal treatment exposure of the study patients. Individual DFPP and TPE procedures and evolocumab administrations are shown according to calendar time. DFPP was temporarily unavailable at our center between December 2021 and November 2022. During this period, Patients 1 and 2 underwent TPE, with evolocumab administered during part of the TPE treatment period. Patient 4 received evolocumab without concomitant apheresis from June to December 2022. DFPP: double-filtration plasmapheresis; TPE: therapeutic plasma exchange.

Generative AI-assisted tools were used solely to assist in the graphical preparation of Figure 2 based on author-provided treatment timeline data. The underlying data, scientific content, and final figure were reviewed and verified by the authors.

## 3. Results

### 3.1. Patient Characteristics and Treatment Exposure

Six patients with HoFH fulfilled the eligibility criteria and were included in the study. Their demographic and clinical characteristics are summarized in Table 1. Genetic testing results were available in four patients and supported the clinical diagnosis of HoFH. Genetic test results were not available in the medical records of the remaining two patients.

During the study period, 476 DFPP procedures were performed. Because DFPP was temporarily unavailable at our center between December 2021 and November 2022, two patients temporarily underwent TPE. Patient 1 underwent six TPE procedures and Patient 2 underwent 14, resulting in a total of 20 TPE procedures. Both patients subsequently resumed DFPP after it became available again. Evolocumab was administered during part of the TPE treatment period in Patients 1 and 2, whereas Patient 4 received evolocumab without concomitant apheresis. The longitudinal treatment course of the individual patients is illustrated in Figure 2.

### 3.2. Changes in Lipid Parameters During Apheresis

Paired pre- and post-procedure biochemical measurements were available for 359 DFPP sessions for total cholesterol, 354 for LDL-C, 357 for HDL-C, and 355 for triglycerides. During DFPP, median total cholesterol decreased from 563 [504–626] mg/dL before the procedure to 194 [157–242] mg/dL after the procedure, corresponding to a median reduction of 64.44% [57.14–70.21]. Median LDL-C decreased from 496.1 [442.8–542.9] mg/dL to 151.6 [120.2–204.3] mg/dL, with a median reduction of 67.53% [59.53–73.86]. Median reductions in HDL-C and triglycerides were 40.00% [30.63–48.00] and 48.63% [35.38–57.66], respectively (Table 2).

Among the 20 TPE procedures performed in two patients, paired pre- and post-procedure measurements were available for 18 procedures for each lipid parameter. Median total cholesterol decreased from 620.5 [581.8–654.3] mg/dL to 190.0 [163.5–202.3] mg/dL, corresponding to a median reduction of 71.11% [65.52–72.69]. Median LDL-C decreased from 522.5 [483.0–552.3] mg/dL to 157.0 [133.8–163.0] mg/dL, with a median reduction of 70.97% [67.14–73.72]. Median reductions in HDL-C and triglycerides were 67.08% [62.92–70.83] and 55.73% [38.68–63.33], respectively (Table 2).

### 3.3. Individual Experience with Temporary TPE and Evolocumab

Patient-level lipid reductions during DFPP and temporary TPE in Patients 1 and 2 are summarized in Table 3. Patient 1 underwent six TPE procedures, of which five had complete paired lipid measurements, whereas Patient 2 underwent 14 TPE procedures, with complete paired measurements available for 13 procedures. No formal statistical comparison between DFPP and TPE was performed because of the limited number of patients exposed to TPE and the repeated nature of the procedures within the same individuals.

Evolocumab was administered during part of the TPE treatment period in Patients 1 and 2. Patient 1 also received evolocumab during a subsequent period without concomitant apheresis, while Patient 4 received evolocumab without concomitant apheresis. Because evolocumab exposure overlapped with part of the TPE treatment period in Patients 1 and 2, the lipid changes observed during these periods could not be attributed solely to the apheresis modality. The longitudinal treatment courses of the individual patients are illustrated in Figure 2.

### 3.4. Safety and Adverse Events

No serious adverse events were observed during DFPP treatment. During temporary TPE treatment, Patient 1 developed chills and subfebrile fever during the sixth TPE procedure, resulting in permanent discontinuation of TPE. Patient 2 did not experience clinically apparent adverse events during TPE. However, an INR of 6.4 was detected in Patient 2, who was receiving long-term warfarin therapy for valvular heart disease. This measurement was obtained before a scheduled TPE procedure, and the INR had already been elevated at 4.44 three weeks earlier. No cardiovascular events or deaths occurred during the temporary TPE treatment period.

## 4. Discussion

In this retrospective, single-centre longitudinal observational study of six patients with HoFH undergoing long-term therapeutic apheresis, DFPP was associated with substantial acute reductions in circulating lipid levels, particularly LDL-C. During a temporary period in which DFPP was unavailable, two patients underwent TPE, which was also associated with marked acute lipid reductions. However, because TPE was administered to only two patients, procedures were repeated within the same individuals, and evolocumab exposure overlapped with part of the TPE treatment period, these findings should not be interpreted as evidence of comparative efficacy between DFPP and TPE.

Despite major advances in pharmacological treatment for HoFH, lipoprotein apheresis remains an important therapeutic option for patients with persistently severe LDL-C elevation despite maximally tolerated lipid-lowering therapy [3,5]. Several selective apheresis techniques are available, including DFPP, cascade filtration, heparin-induced extracorporeal LDL precipitation, direct adsorption of lipoproteins, immunoadsorption, and dextran sulfate adsorption [5,10,19]. Acute LDL-C reduction is an important measure of procedural efficacy, with reductions of approximately 50–70% commonly reported after a single lipoprotein apheresis session [3,5]. In our cohort, the median LDL-C reduction during DFPP was 67.53% [59.53–73.86], whereas the median total cholesterol reduction was 64.44% [57.14–70.21]. These findings are consistent with previous studies reporting substantial acute LDL-C reductions with lipoprotein apheresis [8,12,13,20,21] and with studies specifically evaluating DFPP, in which LDL-C reductions of approximately 64–69% have been reported [22,23,24]. Previous comparative studies of different selective apheresis techniques have also shown marked lipid reductions, although the relative performance of individual modalities has varied according to patient population and procedural characteristics [23,25]. Beyond acute lipid lowering, observational data suggest that regular lipoprotein apheresis may contribute to cardiovascular risk reduction [12,13,14]. Lipoprotein apheresis may also exert pleiotropic effects on inflammatory, oxidative, endothelial, and other pathways involved in atherosclerosis [26].

TPE represents a nonselective plasma-based approach that has historically been used in severe familial hypercholesterolemia when selective lipoprotein apheresis is unavailable or cannot be performed [15,16,17]. Its use in HoFH dates back to the report by Thompson et al. in 1975 [15]. Unlike selective lipoprotein apheresis, TPE removes a broad range of plasma constituents and may therefore reduce not only LDL-C and triglycerides but also HDL-C and other plasma proteins [5,10,17]. In the two patients who temporarily underwent TPE in our study, the median LDL-C reduction was 70.97% [67.14–73.72], while the median reductions in total cholesterol, triglycerides, and HDL-C were 71.11% [65.52–72.69], 55.73% [38.68–63.33], and 67.08% [62.92–70.83], respectively. Although these observations indicate substantial acute lipid removal, the very limited number of patients exposed to TPE and the overlap with concomitant evolocumab therapy preclude conclusions regarding comparative efficacy relative to DFPP.

HDL-C reduction is an expected consequence of both selective lipoprotein apheresis and nonselective plasma exchange, although the magnitude of reduction may vary according to the technique used. Several mechanisms have been proposed for HDL-C loss during lipoprotein apheresis, including hemodilution, filtration-related removal, and alterations in enzymes involved in HDL metabolism. Lipoprotein apheresis may also modify HDL composition and functionality through the removal of apolipoproteins and other associated proteins involved in atherosclerotic risk [5,14,16]. Selective apheresis techniques have generally been reported to produce smaller reductions in HDL-C than nonselective plasma exchange, although substantial variability exists among individual modalities and studies [21,22,23,27]. In our cohort, the median HDL-C reduction was 40.00% [30.63–48.00] during DFPP and 67.08% [62.92–70.83] during TPE. Given that TPE was administered to only two patients, these values should not be interpreted as evidence of a statistically or clinically meaningful difference between the modalities. The observed pattern is nevertheless consistent with the nonselective nature of TPE and should be considered when interpreting its temporary use if selective lipoprotein apheresis is unavailable.

Although apheresis produces substantial acute reductions in LDL-C, its effect is transient, and sustained lipid lowering generally requires concomitant pharmacological therapy [3]. PCSK9 inhibitors, including evolocumab and alirocumab, reduce circulating LDL-C by increasing hepatic LDL receptor availability. Their efficacy in HoFH is strongly influenced by residual LDL receptor function and is therefore more variable than in heterozygous familial hypercholesterolemia [3,6,28,29]. Previous studies of evolocumab in HoFH have reported modest and heterogeneous LDL-C reductions [28,29], whereas larger reductions have generally been reported in HeFH populations [30,31]. In our cohort, evolocumab was administered to three patients. In Patients 1 and 2, treatment overlapped with part of the temporary TPE period, precluding reliable attribution of subsequent LDL-C changes to evolocumab alone. Patient 4 received evolocumab without concomitant apheresis but did not show a clear LDL-C reduction during the observed treatment period. Given the known dependence of PCSK9 inhibitor efficacy on residual LDL receptor function in HoFH, interindividual variation in LDL receptor function may have contributed to the observed responses; however, functional LDL receptor activity was not assessed in our patients.

Adverse events associated with lipoprotein apheresis are generally uncommon but may include hypotension, allergic reactions, nausea, anemia, and vascular access-related complications [5,10,16]. TPE, as a nonselective procedure, may additionally affect coagulation factors and is associated with bleeding, infection, electrolyte disturbances, and reactions related to replacement fluids [17]. In our cohort, no serious adverse events were observed during DFPP. During temporary TPE treatment, Patient 1 developed chills and subfebrile fever during the sixth procedure, leading to permanent discontinuation of TPE. Patient 2 did not experience clinically apparent adverse events during TPE. However, this patient, who was receiving long-term warfarin therapy for valvular heart disease, had an INR of 6.4 during the treatment period. Importantly, the INR measurement was obtained before the scheduled TPE procedure, and the INR had already been elevated at 4.44 three weeks earlier. Although TPE may affect coagulation parameters in patients receiving warfarin [32], the temporal pattern in our patient does not support attribution of the INR elevation to TPE. Given the very limited number of patients exposed to TPE, our observations do not allow conclusions regarding its safety relative to DFPP.

The main limitations of this study are its retrospective, single-center design and the small number of individual patients. Although a large number of apheresis procedures were evaluated, repeated procedures were performed within the same individuals and therefore did not represent statistically independent observations. TPE was administered to only two patients, precluding formal comparative efficacy or safety analyses between TPE and DFPP. In addition, evolocumab treatment overlapped with part of the TPE period, introducing potential confounding in the interpretation of lipid changes. Genetic data were not uniformly available, functional LDL receptor activity was not assessed, and some biochemical measurements were missing. The absence of standardized long-term cardiovascular outcome data further limits interpretation. Finally, the single-center setting may reduce generalizability to healthcare systems with different access to apheresis technologies.

Despite these limitations, the study provides longitudinal real-world data on long-term DFPP treatment and the temporary use of TPE in HoFH, a rare disorder for which long-term real-world treatment data remain limited. The extended observation period and the availability of paired pre- and post-procedure lipid measurements across a large number of apheresis procedures strengthen the longitudinal assessment. In addition, individual treatment histories provide useful clinical context for interpreting changes in apheresis modality and concomitant therapies.

## 5. Conclusions

Long-term DFPP was associated with substantial acute reductions in LDL-C and other lipid parameters in patients with HoFH. During the temporary interruption of access to selective lipoprotein apheresis, TPE also produced marked acute lipid reductions in two patients. However, the limited number of patients exposed to TPE, the repeated procedures within the same individuals, and the overlap with evolocumab treatment precludes conclusions regarding comparative efficacy or safety between DFPP and TPE. TPE may be considered as an individualized interim option when selective lipoprotein apheresis is temporarily unavailable. Further evidence is needed to clarify its role in this setting.

## Figures and Tables

**Table 1:** Demographic and clinical characteristics of the study patients.

Patient	Sex	Age, Years	Age at Diagnosis, Years	Age at Regular LA Initiation, Years	Main Presentation	Major Cardiovascular History	Comorbidities/Risk Factors	Family History	Genetic Testing	Baseline LDL-C, mg/dL	Last Status
1	M	30	6	20	Xanthoma	Mild AS (16)	Multiple sclerosis	Father died from MI at age 55	Available	447	LA ongoing
2	F	51	7	39	Xanthoma	MAVR and CABG (34), CS (39)	–	Father	Available	583	LA ongoing
3	M	27	9	23	Xanthoma	CABG and CEA (23)	Smoking, AH	Brother	Available	612	LA ongoing
4	M	41	20	37	Xanthoma	MAVR and CABG (31)	MRH, Smoking	Mother and sister	Available	532	LA discontinued
5	M	32	6	11	Xanthoma	CABG (12), CAS (31), AS (31)	–	Unknown	Not available in medical records	598	Died at age 32
6	M	49	27	43	ACS, xanthoma, XP	CABG (27), CEA (38), CS (45)	AH	Sister	Not available in medical records	543	LA discontinued

Baseline LDL-C refers to the LDL-C level before the initiation of regular lipoprotein apheresis. ACS: acute coronary syndrome; AH: arterial hypertension; AS: aortic stenosis; CABG: coronary artery bypass graft; CAS: carotid artery stenting; CEA: carotid endarterectomy; CS: coronary stent; F: female; LA: lipid apheresis; M: male; MAVR: metallic aortic valve replacement; MI: myocardial infarction; MRH: multicentric reticulohistiocytosis; XP: xanthelasma palpebrarum. Major cardiovascular history: numbers indicate the age at which the event occurred.

**Table 2:** Pre- and post-procedure lipid levels during double-filtration plasmapheresis and temporary therapeutic plasma exchange.

Apheresis Modality	Parameter	Paired Sessions, *n*	Pre-Procedure, Median [Q1–Q3] (mg/dL)	Post-Procedure, Median [Q1–Q3] (mg/dL)	Reduction, %, Median [Q1–Q3]
DFPP	Total cholesterol	359	563 [504–626]	194 [157–242]	64.44 [57.14–70.21]
DFPP	LDL-C	354	496.1 [442.8–542.9]	151.6 [120.2–204.3]	67.53 [59.53–73.86]
DFPP	HDL-C	357	37 [31–43]	22 [20–26]	40.00 [30.63–48.00]
DFPP	Triglycerides	355	146 [121–184]	76 [57–104]	48.63 [35.38–57.66]
TPE	Total cholesterol	18	620.5 [581.8–654.3]	190.0 [163.5–202.3]	71.11 [65.52–72.69]
TPE	LDL-C	18	522.5 [483.0–552.3]	157.0 [133.8–163.0]	70.97 [67.14–73.72]
TPE	HDL-C	18	48.0 [42.0–51.3]	15.5 [14.0–17.0]	67.08 [62.92–70.83]
TPE	Triglycerides	18	200.5 [154.5–241.8]	85.5 [66.5–113.5]	55.73 [38.68–63.33]

Data are presented as median [Q1–Q3]. Only sessions with paired pre- and post-procedure measurements for the relevant lipid parameter were included. DFPP: double-filtration plasmapheresis; TPE: therapeutic plasma exchange; LDL-C: low-density lipoprotein cholesterol; HDL-C: high-density lipoprotein cholesterol; Q1, first quartile; Q3, third quartile.

**Table 3:** Individual lipid reductions during DFPP and temporary TPE in Patients 1 and 2.

Patient	Modality	*n*	TC Reduction, %	LDL-C Reduction, %	HDL-C Reduction, %	TG Reduction, %
1	DFPP	63	64.84 [61.26–67.04]	68.17 [63.61–70.33]	42.86 [37.93–48.94]	45.38 [32.12–58.06]
1	TPE	5	65.18 [63.50–65.48]	65.88 [61.53–67.01]	61.90 [58.51–65.15]	38.85 [34.88–55.37]
2	DFPP	162	69.86 [65.41–73.78]	73.84 [69.18–77.19]	44.44 [21.24–50.00]	50.19 [37.68–58.37]
2	TPE	13	71.88 [70.65–73.50]	72.03 [70.78–75.99]	69.39 [65.69–70.97]	60.67 [47.78–65.95]

Data are presented as median [Q1–Q3]. Only sessions with paired pre- and post-procedure measurements were included. DFPP: double-filtration plasmapheresis; TPE: therapeutic plasma exchange; TC: total cholesterol; TG: triglycerides.

## Data Availability

The datasets analysed during the current study are available from the corresponding author upon reasonable request.
